# Watching risk: fairness, medical supervision, and the spectator problem in the enhanced games

**DOI:** 10.3389/fspor.2026.1843832

**Published:** 2026-09-08

**Authors:** Alexis Demas

**Affiliations:** ARGOS (Art, Research and Gestures Observed Scientifically), Neurosciences and Arts, Paris, France

**Keywords:** autonomy, doping, enhanced games, fairness, spectatorship

## Abstract

The Enhanced Games are often presented as an ethically improved alternative to conventional anti-doping sport because they replace prohibition with transparency, athlete autonomy, and medical supervision. The inaugural event, held in Las Vegas in May 2026, complicates rather than confirms this framing: several winners competed without enhancement, the substances used by others were often undisclosed, and only one world record was set. Open enhancement therefore does not straightforwardly resolve the ethical problems of fairness or harm; it reorganizes competition around access to biomedical optimization, tolerance of pharmacological risk, and physician-enabled performance escalation. This model also raises a neglected question: the ethics of the spectator. If audiences knowingly consume performances built on deliberate pharmacological risk, the moral problem is no longer confined to the athlete's consent; it extends to the collective normalization of medically hazardous enhancement as entertainment. The Enhanced Games should therefore be assessed not because they introduce risk into an otherwise safe world of sport, but because they institutionalize enhancement-related risk as a structural feature of competition and convert that risk into spectacle, with spectators as active, differentiated participants in that conversion.

## Introduction

The Enhanced Games launched their promotional framing well before their first event, describing an arrangement in which athletes compete under “strict, clinical and medical supervision,” with athlete “autonomy and choice” repeatedly foregrounded (1). The inaugural Games took place on 24 May 2026 in Las Vegas, and the record now allows these claims to be checked against practice rather than assessed on promise alone. The results complicate the organization's framing more than they confirm it. Only one of roughly twenty-two events produced a ratified-standard-beating performance; three event winners, including the men's and women's 100 m sprint champions, competed without enhancement to preserve Olympic eligibility; and the specific substances used by enhanced competitors were often not disclosed. An independent commentary in the BMJ concluded that the event's claims about safety and performance rest on weak evidence (2). None of this shows that medical supervision is absent, but it does show that “strict, clinical and medical supervision” is, at this stage, an organizational claim rather than an independently verified practice, and that predictions about records, safety, and reach should be treated as such.

This matters because the ethical debate about the Enhanced Games often collapses into a binary: prohibition versus open regulation. That binary is too coarse. The case against the Games is weak if it depends on pretending that ordinary elite sport is already a realm of purity and equal opportunity; it is not (3). The stronger claim is that the Games do not merely tolerate risk, as conventional sport already does, but redefine competition through the organized medical management of pharmacological risk, and that this redefinition creates a distinct ethical problem at the level of the audience that consumes it.

## Engaging the strongest case for enhancement

Proponents make four serious arguments. Savulescu and colleagues argue that enhancement can compensate for the genetic lottery and thereby promote fairness (4). Others argue that prohibition has failed and driven doping underground, that enhancement merely extends existing forms of optimization such as altitude training and biomedical monitoring, and that competent adults should be free to accept understood risks (5). Ekdahl and Krieger (6) add a further, more institutional version of this case: the Enhanced Games position themselves not merely as a doping-tolerant event but as a more economically and ecologically sustainable, athlete-centred alternative to the Olympic model, which deserves engagement on its own terms rather than dismissal as a doping spectacle alone.

These arguments have force, but the inaugural Games weaken two of them empirically. The fairness-through-enhancement argument presupposes roughly equal access to enhancement technology; instead, access to safer, more sophisticated, physician-managed protocols remains stratified by resources, so enhancement is more likely to layer a biomedical hierarchy onto existing advantage than to remove one. The harm-reduction argument presupposes that supervision is transparent; the undisclosed substances at the inaugural event suggest that supervision can coexist with exactly the opacity it is meant to replace. The sustainability and athlete-centred case advanced by Ekdahl and Krieger deserves more credit: it is not refuted by one event, but it also does not, by itself, answer the fairness and spectator questions developed below.

## Autonomy under structural pressure

Athlete autonomy is ethically important but insufficient. Athletes do not choose enhancement in a social vacuum; ranking pressure, sponsorship exposure, and career insecurity shape what counts as a reasonable choice, and once enhancement is structurally available and financially rewarded, the decision an athlete faces is no longer simply whether to accept risk but whether to accept competitive displacement by those who do (7). Hyde (8) develops the specific bioethical version of this concern for the Enhanced Games: a $1 million record bonus and salaried contracts function as the kind of payment that bioethics already treats as a potential coercive offer in research participation, and the same logic weakens consent here. The concern is not that athletes are explicitly forced to enhance; it is that competitive systems reshape what a reasonable choice looks like.

## Fairness: which principle, and why biomedical risk differs

Critics of anti-doping correctly note that elite sport already distributes genetics, coaching, and equipment unequally. But this observation equivocates between distinct principles. Equality of access to training resources is not equality of opportunity, and neither is equality of exposure to health risk. Unequal coaching or altitude facilities create unequal preparation; they do not require an athlete to accept probabilistic, cumulative, sometimes irreversible harm to their body as a precondition of competing at all (5, 9, 10). Biomedical inequality is therefore not simply one more axis of an already unequal sport. It converts competitive disadvantage into a health-risk asymmetry, which is a different kind of unfairness and a graver one.

## Medical supervision: legitimacy is not the same as safety

Medical supervision should not be caricatured, but neither should it be treated as ethical absolution (11, 12). Physicians in enhancement protocols occupy a structural conflict: they may act simultaneously as protectors of athlete health and enablers of performance escalation. A useful, imperfect analogy is boxing, where physician presence at ringside has been argued not to resolve the moral problem of a sport built around foreseeable harm but to lend it medical legitimacy (13). Wiesing (14) makes the underlying question explicit: legalizing enhancement under medical supervision does not by itself answer whether medicine should lend its authority to non-therapeutic, performance-driven pharmacology. The inaugural Games’ undisclosed substances suggest that, in practice, supervision has so far functioned at least as much as public reassurance as verified protection.

## The ethics of the spectator

The spectator problem needs to be separated into three distinct claims, which the literature on fan ethics allows us to distinguish rather than conflate.

The first is complicity. Spectators do not prescribe protocols or administer drugs, but their attention, subscriptions, ticket purchases, and bets constitute the economic and symbolic value the event sells. Complicity is not homogeneous: watching a free highlight clip is a weak, largely non-contributory form of exposure, whereas buying a ticket, subscribing, or betting directly funds the next edition and therefore carries a heavier causal and moral weight. A defensible spectator ethics has to track this gradient rather than treat “the audience” as one undifferentiated actor.

The second is normalization through what Smith and Lord (15) call bracketed morality: sport fandom already involves a suspension of everyday ethical standards, in which spectators accept behaviour, from partisan hostility to celebrated risk-taking, that they would not accept outside the sporting frame. The Enhanced Games extend this bracket to include pharmacological risk itself: the object of admiration is no longer only skill but chemically intensified performance, and bracketed morality supplies the psychological mechanism by which spectators can enjoy this without registering themselves as implicated.

The third is moral pedagogy: elite sport communicates, beyond any individual spectator's attitude, what a society collectively chooses to admire. Whether this pedagogy measurably shapes younger athletes’ own attitudes toward enhancement, specifically through exposure to elite spectacle, is not yet established by direct evidence; existing doping-prevention research addresses the efficacy of educational interventions aimed at young athletes (16) rather than the normalizing effect of spectatorship itself, and the claim should be treated as a plausible, testable hypothesis rather than a settled finding.

Taken together, these three mechanisms do not require that every spectator be individually blamed for an athlete's future disease. They require recognizing that collective, differentiated attention is part of the mechanism by which medically hazardous enhancement becomes intelligible, fundable, and repeatable as spectacle, and that an ethics of the Enhanced Games which stops at the athlete and the physician has not yet reached the level at which the event is sustained.

## Conclusion

This argument builds on an earlier assessment of the Enhanced Games as a redefinition of athletic values, but its contribution here is different and more specific: a sustained, differentiated account of spectator responsibility as an independent locus of ethical evaluation, tested against the inaugural event rather than its promotional claims. Athlete autonomy deserves respect, but it does not settle the question; fairness is not rescued when inequality becomes biomedical; supervision is not the same as safety; and spectatorship is not innocent when public attention, unevenly and knowingly given, helps fund performances built on possible long-term harm. Anti-doping education, youth-sport policy, and Olympic governance would be well advised to treat the Enhanced Games as more than a marginal experiment, though establishing that they constitute a lasting cultural turning point will require evidence from further editions, not this one alone.
